# New Percutaneous Options for Tricuspid Intervention: How to Identify the Good Clinical Candidate

**DOI:** 10.3389/fcvm.2020.583307

**Published:** 2020-12-21

**Authors:** Alessandra Laricchia, Arif A. Khokhar, Francesco Giannini

**Affiliations:** Interventional Cardiology Unit, GVM Care & Research Maria Cecilia Hospital, Cotignola, Italy

**Keywords:** timing, operative risk, surgery, tricuspid regurgitation, percutaneous (catheter-based) treatment

## Abstract

The tricuspid valve has been neglected for a long time and severe tricuspid regurgitation (TR) was largely undertreated in the past due to a high operative risk. In the last years we observed the development of different less invasive percutaneous options to treat TR. Currently, percutaneous treatments are reserved for high-risk patients presenting with advanced stage disease by which time they are likely to derive a partial benefit at best. There is a limited evidence base, including no randomized trials, to guide the management strategy for severe TR. In the interim we feel that choosing the best device for the most appropriate clinical candidate and with an adequate timing (most probably an “earlier” timing) will be the key combination to improve early and late outcomes of percutaneous treatments.

## Introduction

Tricuspid regurgitation (TR), a common finding in the elderly population, is more frequently caused by left sided heart disease or pulmonary hypertension (functional), whilst primary and isolated TR are less frequent (1).

The natural history of TR is difficult to predict, with the temporal evolution from mild to significant TR being variable. Furthermore, the degree of functional TR is highly dependent upon right ventricular (RV) preload (i.e., intravascular volume status), afterload and RV systolic function and severe TR may be masked by the absence of symptoms (2). Most importantly, severe TR is associated with poor outcomes as patients on medical therapy alone, have a 63.9% rate of event free survival at 1.4 ± 1 years (3).

In spite of this, only a small proportion of patients undergo surgical TV repair or replacement. This can partially be ascribed to the wrong belief that correcting the primary disease (like left heart valve disease) may lead to an improvement in secondary TR (4, 5). In addition, a high operative risk may preclude many patients from surgical treatment. However, the reported rate of in-hospital mortality following TR surgery is highly variable amongst different series (10–38% of in-hospital mortality). Delayed referrals may account for this variability, thus highlighting the importance of optimal timing for intervention (6, 7).

Accordingly, in 2017, guidelines were updated to recommend surgery for mild or moderate TR associated with left sided valve surgery, if the annulus is dilated or in the presence of heart failure (8, 9). However, these class IIa/IIb recommendations (level of evidence C) are supported by a limited evidence base and lack of randomized trials comparing surgery vs. medical therapy. Moreover, isolated TR is excluded from these recommendations with only severe symptomatic TR or with initial right ventricle dysfunction being an indication for surgery.

The role of optimal medical therapy is not addressed by guidelines. Whilst diuretics provide symptoms relief, ACE inhibitors or beta-blockers have not shown any prognostic benefit in right heart disease.

Subsequently, determining the optimal management strategy for these patients is complex. In the last few years, the development of less invasive percutaneous treatment options offers new strategies for high-risk or inoperable patients. They are not included in current guidelines as their role in the management of severe TR still needs to be fully elucidated. The purpose of this review is to highlight the potential clinical applications of these therapies and to identify which patients are likely to derive the most benefit.

## Current Role of Percutaneous Tricuspid Interventions

Despite the clear relationship between severe TR and mortality, surgical treatment is only offered to a small group of patients. This is due to the significant in-hospital mortality of these patients who often have advanced right ventricular dysfunction with multiple coexisting comorbidities. Subsequently, several less invasive percutaneous repair and replacement treatment strategies have been developed for this undertreated high-risk cohort (10). In the last few years, first-in-human reports, compassionate clinical programs and early feasibility studies for transcatheter tricuspid valve therapies (TTVT) have been published (11–18). However, these transcatheter treatments have been reserved for patients with a high-risk profile presenting at an advanced stage of their disease (19). Moreover, there are no randomized studies comparing TTVT vs. optimal medical therapy. Additionally, the small number of patients enrolled in these studies, limits the ability to verify the real clinical impact of these procedures on clinical outcomes. Data gathered from various registries of patients treated with different devices, can partially overcome these limitations and have reported encouraging results. Firstly, percutaneous TV intervention has a high overall procedural success rate (≈90%) that is associated with greater survival and reduced heart failure hospitalizations compared to optimal medical therapy alone, regardless of TR severity, NYHA class, and RV dysfunction at baseline (20). Secondly, a low rate of procedure-related complications (2% of conversion to open heart surgery) as well as an average 30-day mortality rate of 5.1% compare favorably with isolated TV surgery (6, 21). However, the definition of procedural success used in the aforementioned studies (≥1 TR grade reduction) has some limitations, given the absence of control groups for comparison as well as of data about prognosis (22). In addition, the marked improvement in functional status (evaluated as NYHA functional class or 6 min walking distance) and quality of life indices is hardly explained if only a modest reduction in TR degree is usually achieved, thus with a potential for placebo effect. Accordingly, further validation of TTVT in larger cohorts and randomized trials with prolonged follow-up is now required prior to expand indications and potential guideline recommendations.

At this purpose, choosing the right device for the right patient at the right time is crucial to ensure both procedural success and beneficial acute and long-term clinical outcomes.

## The Good Clinical Candidate

Considering the complexity of TV anatomy, the multiple mechanisms involved in TR and their potential variation throughout the disease course, consideration for TTVT requires a multidisciplinary Heart Team evaluation of the potential risks, expected benefits, anatomical suitability, and technical issues.

**Figure 1:**
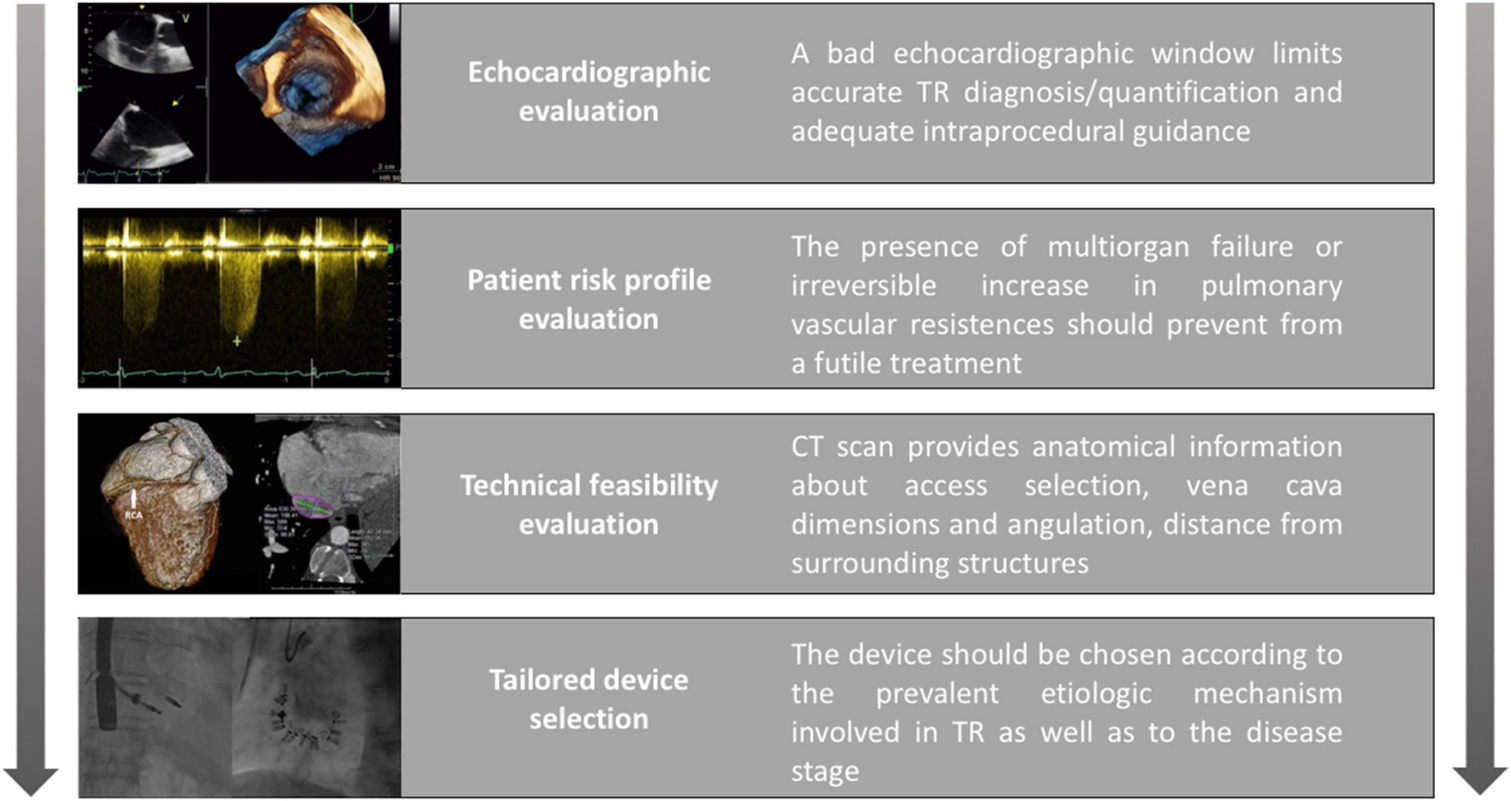
Step-by-step approach to identify the good clinical candidate.

We highlight four main steps in the decision-making process (Figure 1):

- First, availability of good quality imaging is critical for pre-procedural planning and intraprocedural guidance. Transesophageal echocardiography focused on the TV is essential and should be performed by a trained physician familiar with special views (such as deep trans-esophageal and trans-gastric short axis views) and three-dimensional (3D) reconstructions, which can aid visualization and comprehension of the complex TV anatomy (23).Nevertheless, TV imaging can be challenging and when echocardiographic windows are suboptimal, the transcatheter option should be reconsidered. The presence of other prosthetic devices (including valves and electronic devices) can generate artifacts that can potentially worsen the image quality. An emerging alternative for intraprocedural monitoring is intracardiac echocardiography (ICE). However, its application in clinical practice is currently limited due to poor expertise and technical limitations such as the absence of multiplanar views. The latter makes procedural guidance impossible for Clips and Cardioband. Development of the ICE technology to incorporate 3D visualization may overcome this limitation.
- Secondly, assessment of a patient's global status and risk profile is based on a combined clinical and instrumental evaluation. The presence of significant comorbidities which reduce life expectancy, can limit the potential benefit of any interventional treatment. Similarly, correcting TR when the disease is too advanced could be futile. Clinically, in the late stages of the disease the presence of organ damage (including liver and kidney dysfunction) due to severe venous congestion as well as the presence of symptoms and signs of low cardiac output (such as fatigue, asthenia, and poor functional capacity) should be excluded as they correlate with poorer outcomes (24). Furthermore, the assessment of right ventricular (RV) function and pulmonary vascular status should be also performed. Correction of TR in a severely dysfunctional RV may precipitate acute right heart failure due to the sudden afterload mismatch. RV remodeling is a distinctive feature of pathological TR which occurs either in primary TR to accommodate volume overload or in secondary TR to overcome pressure overload associated with pulmonary hypertension. It usually starts as an adaptive mechanism to maintain cardiac output but subsequently transforms into a maladaptive response resulting in RV dysfunction. However, interpreting the echocardiographic parameters of RV function (tricuspid annular plane systolic excursion [TAPSE], tissue doppler S', fractional area change [FAC]) is challenging in the setting of severe TR as they are largely influenced by loading conditions (25). Moreover, the pulmonary artery systolic pressures (PASP) estimated from echocardiography can be misleadingly low when the stroke volume is reduced. Additionally, independently evaluating ventricular and pulmonary function is difficult given the strict dependency of RV performance on its afterload. To overcome this limitation, right heart catheterization (RHC) should be considered. In addition to directly measure the pulmonary pressure, pulmonary vascular resistance (PVR) can be determined which reflects pulmonary vascular remodeling. Furthermore, the RV systolic performance at a given degree of afterload (named right ventricular to pulmonary artery [RV-PA] coupling) can be measured by analyzing the pressure-volume loops. The ventricular-vascular coupling ratio is a variable which quantifies the interaction between ventricular contractility and vascular afterload providing an additional measure of cardiovascular efficiency (26). Recently, non-invasive MRI and echocardiographic surrogates of RV-PA coupling have been also proposed (27, 28).
- Thirdly, anatomical evaluation combining echocardiography with computed tomography (CT) to assess technical feasibility and procedural strategy is critical. Important anatomical considerations include:
  ° the size and angulation of the superior and inferior vena cava with respects to the TV apparatus to select the best vascular access.
  ° the space available for device navigation in the right chambers,
  ° landing zone geometry,
  ° identification of surrounding structures, especially the course of the right coronary artery (RCA), which can potentially be damaged during the procedure.
- Finally, an appropriate device should be selected, with considerations given to the underlying etiology as well as the stage of the disease. An easy algorithm that was previously proposed distinguishes between primary and secondary etiologies (21). For primary TR, valvular replacement is the preferred strategy especially for rheumatic etiologies. There is limited literature in the form of case-reports demonstrating the successful use of MitraClip (Abbott Vascular, Santa Clara, California) for leaflet prolapse or lead-induced TR in the absence of extreme annular dilatation (29, 30). Conversely, secondary or functional TR in its earlier stages can be easily addressed through annuloplasty strategies. As the disease progresses and with increasing leaflet tethering, edge-to-edge devices either alone or in combination with annuloplasty systems for a synergistic effect may be the preferred treatment strategy. If RV function is preserved, then orthotopic valve replacement can also be considered (31), otherwise if RV remodeling is extensive then heterotopic valve implantation is preferred as a palliative strategy in selected cases (32, 33).

## The Good Device

When selecting the appropriate device, both the underlying etiology and specific anatomic criteria should be considered.

A brief summary of available devices and their correct application is illustrated in Table 1. Below we provide a more detailed description about the anatomic features that are associated with a successful procedure for the different devices.

**Table 1:** Device selection based on the etiologic mechanism involved in TR as well as on specific anatomic features.

		**Device mechanism of action**			
		**Annuloplasty**	**Edge-to-Edge Repair**	**Etherotopic Replacement**	**Orthotopic Replacement**
		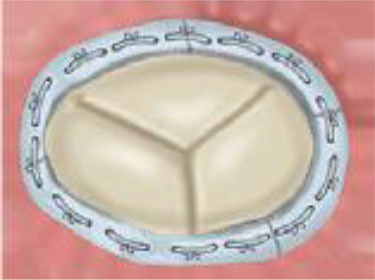	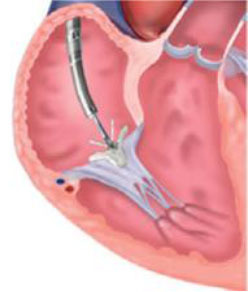	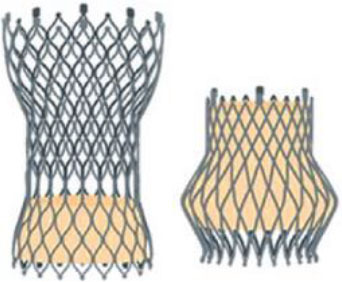	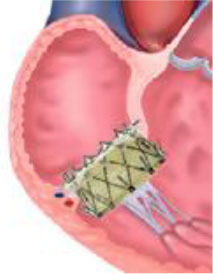
		Ideal for AP diameter <36 mm and tenting volume <1.68 mL	Ideal for coaptation gap <7 mm	Palliative symptomatic for advanced disease	Ideal for functioning RV even for larger annuli and coaptation gap
Primary TR	Leaflet prolapse	NOT INDICATED	CAN BE USED (22, 23)	NOT INDICATED	NOT INDICATED
	Lead interference	CAN BE USED (34)	CAN BE USED (34)	NOT INDICATED	CAN BE USED (35)
	Rheumatic	NOT INDICATED	NOT INDICATED	NOT INDICATED	CAN BE USED
Secondary TR	Annular dilatation	INDICATED (27)	COMBINATION (28)	NOT INDICATED	CAN BE USED (24)
	Leaflet tethering	COMBINATION (28)	INDICATED (29)	NOT INDICATED	CAN BE USED (24)
	RV remodeling	NOT INDICATED	NOT INDICATED	CAN BE USED (25)	NOT INDICATED

Annuloplasty is designed to reduce annular dimensions and promote leaflet coaptation and is most effective in the earlier stages of the disease process. Based on surgical experience, percutaneous ring-based systems provide more effective and durable results in comparison to suture-based systems. As annular dilatation worsens, there is a progressive decrease in leaflet coaptation. Tenting volumes of more than 1.68 mL as well as antero-posterior annular diameters larger than 36 mm are predictive for persistent or recurrent TR after annuloplasty (36). In these circumstances combined repair strategies should be considered and some successful experiences have been reported (37). For larger annuli, a valve replacement strategy may be more effective. The NaviGate (NaviGate Cardiac Structures) self-expanding bioprosthesis is available in four sizes which are suitable for annular diameters ranging from 36 to 52 mm, with minimal oversizing recommended (31).

Different technologies mimicking the edge-to-edge surgical repair are currently available. So far, the MitraClip has been the most frequently used device to correct TR and therefore has the largest evidence base.

Besler et al. analyzed data from 117 patients treated by percutaneous edge-to-edge repair and found that a smaller effective regurgitant orifice area (EROA), tenting area, vena contracta (VC) and coaptation gap together with a central/anteroseptal jet location were predictors of procedural success at univariate analysis. In the multivariate model only the last 2 factors independently predicted transcatheter TR repair success. The proposed cut-off values for the univariate predictors were as follows: 0.6 cm^2^ for EROA, 2.1 cm^2^ for tenting area and 11 mm for VC. Conversely, a cut-off value for a coaptation gap of 7.2 mm was identified as the best discriminator for successful repair. Treatment success declined linearly with the magnitude of the coaptation gap, yielding a success rate of <30% with a gap of more than 10 mm. Interestingly, none of the patients with a combined coaptation gap of more than 7.2 mm and non-central/non-anteroseptal jet location achieved procedural success (38). The authors concluded that a larger coaptation gap hinders successful clip placement or can lead to clip placement in a wrong position far from the main regurgitant jet. Furthermore, a non-central/non-anteroseptal regurgitant jet location reflects a more technically challenging procedure.

An *ex-vivo* experimental model of functional TR found that anteroseptal clipping was more effective in reducing TR compared to posteroseptal grasping or anteroposterior grasping, which is often ineffective and occasionally detrimental (34). Clip placement in the anteroseptal and posteroseptal coaptation lines may counteract the outward pulling forces of the annulus, whilst clipping along the anteroposterior line may worsen the coaptation with the septal leaflet by bringing the coaptation line between the anterior and posterior leaflets under tension (35).

More recently, the development of the new MitraClip XTR, with its 3 mm longer arms in comparison to its predecessor NTR, has opened the possibility to treat larger coaptation gaps. Braun et al. reported the results from 31 patients treated with MitraClip XTR for functional TR, amongst whom 16 patients (52%) had a coaptation gap ≥ 7 mm. Procedural success was achieved in 87% of total cases and in 75% of those with larger gaps, with a 30-day residual TR ≤ 2 of 69 and 43%, respectively. Of note, they observed a significant rate of single leaflet clip detachment in patients with coaptation gap ≥ 7 mm (3 in hospital and 1 later on) (39). Thus, the implantation of larger clips in wider coaptation gaps does not guarantee improved outcomes. On the contrary, it may lead to incomplete leaflet insertion and possibly laceration. Given that TV leaflets are usually thin and fragile, longer leaflets would be more suitable for the larger XTR clip as the portion of the clip that holds the leaflet with the greatest force corresponds to its most proximal segment. Alternatively, implantation of a second smaller NTR clip adjacent to the therapeutic XTR clip may promote stability, decrease the tension on the leaflets and reduce the probability of laceration (40). Nevertheless this possibility needs to be validated in larger studies.

The Pascal (Edwards Lifesciences, Irvine, California) system is another percutaneous edge-to-edge repair system similar to the MitraClip. Its potential advantages are the possibility of independent leaflet capture, which can facilitate grasping, as well as the central spacer, which may improve TR correction in the most severe cases (41). However, in a recent compassionate use experience, despite an effective reduction of TR, a low but not negligible incidence of leaflet detachment was still observed (7%, 2 out of 28 patients) (14).

Less data exists regarding percutaneous treatment of TR in the presence of cardiac implantable electrical devices (CIED). CIED are present in up to 1/3 of patients undergoing transcatheter treatment for functional TR and device leads are themselves a frequent cause of significant TR (19, 42). The presence of a pacemaker lead is one of the clinical predictors of recurrent TR after surgical annuloplasty, mainly because of residual leaflet or subvalvular impingement (24). Moreover, the presence of a lead poses specific technical challenges in terms of procedural imaging and device positioning. Updated findings from the TriValve registry on 470 patients comparing CIED (26%) against non-CIED patients (74%) found comparable feasibility, acute procedural success, safety, and short-term outcomes between the two groups (43). Thus, the presence of a pacemaker lead through the TV should not preclude a transcatheter option to fix TR. When faced with both an atrial and a ventricular lead, it's important to place the guiding catheter and the delivery system in between or medial to the leads to allow sufficient space to maneuver the device in the right atrium and avoid entanglement. One strategy, which can be considered with edge-to-edge repair systems is the bicuspidalization of opposing leaflets and/or isolation of the pacemaker lead.

Of note, the large majority of patients in the aforementioned series were treated by MitraClip implantation (87.6%), thus making it difficult to infer conclusions about different devices, and the procedures were performed in highly experienced centers.

In addition, percutaneous TV replacement also appears to be feasible in patients with CIED. The risk of lead damage during valve-in-valve procedures, due to outward displacement and entrapment of the leads between the new and the native valve, appears to be negligible (44).

## Optimal Timing for Intervention

Transcatheter treatments for TR have been developed for patients with advanced heart failure and multiple comorbidities. Subsequently, the primary aim of these therapies is to provide symptomatic relief as opposed to curative management as achieved in percutaneous aortic or mitral valve interventions. Nevertheless, identifying the “optimal timing” for intervention is essential to avoid futility. For advanced stages of the disease, palliative strategies such as compassionate use of heterotopic or orthotopic valve implantation can in selected cases, reduce central venous congestion, and enhance the efficacy of medical therapy. Conversely, complete correction of TR in patients with severe RV dysfunction can lead to sudden afterload mismatch and precipitate acute heart failure. Nevertheless, the measurement of RV function is complex, due to its unique geometry and its dependence upon pre-load and after-load. A single index with its own cut-off value can be of limited value, whilst a multiparametric assessment utilizing multiple imaging modalities (including echo and MRI) will better capture the complexity of RV structure and function in the context of severe TR (25, 45).

Given that higher procedural success and improved durability were observed in patients with smaller annuli and coaptation gaps, “optimal timing” could be translated into “earlier timing.” If utilized earlier in the disease process, these technologies could eventually promote reverse remodeling of the RV and TV apparatus (46). Furthermore, minimizing TR early on may halt the disease process from progressing, which may ultimately translate into long-term prognostic benefit for the patients.

## Future Perspectives and Conclusions

The experience with percutaneous treatment of TR is in its initial stages with limited experience. Key lessons learnt include the critical impact of patient/device selection according to specific anatomic features and timing of intervention, on early and late clinical outcomes. Further improvements in device technology, imaging solutions, and procedural performance are still required.

New insights on the topic are eagerly awaited from the first randomized trials comparing tricuspid valve repair to optimal medical therapy (OMT). The CLASP II TR study will compare transcatheter tricuspid valve repair with the Edwards PASCAL Transcatheter Valve Repair System plus OMT to OMT alone (NCT04097145), whilst the TRILUMINATE trial will compare the Abbott TriClip System to OMT (NCT03904147).

In the near future, TTVT may be required to treat a growing new category of patients, those undergoing percutaneous left sided interventions. Residual TR after mitral or aortic valve treatment is associated with longer and more complicated hospitalizations, heart failure readmissions and mortality (47, 48). A combined percutaneous edge-to-edge procedure on both the mitral and tricuspid valve is superior to isolated mitral valve repair in terms of functional improvement early after the intervention and clinical outcomes at follow-up (49).

Given the high prevalence of multivalvular disease, the expanding indications for transcatheter aortic valve implantation (TAVI) and the widespread use of percutaneous treatment for the mitral valve, the future holds the possibility to offer a complete transcatheter treatment with results comparable to those achieved by surgery.

## Author Contributions

All authors have equally contributed to the ideation of this work and have reviewed and agree with the content of the article.

## Conflict of Interest

The authors declare that the research was conducted in the absence of any commercial or financial relationships that could be construed as a potential conflict of interest.
